# Efficiency and Privacy in Record Linkage: Evaluating a Novel Blocking Technique Implemented on Cryptographic Longterm Keys

**DOI:** 10.23889/ijpds.v9i5.2534

**Published:** 2024-09-18

**Authors:** Dean Resnick, Núria Adell Raventós

**Affiliations:** 1 NORC at the University of Chicago

**Keywords:** Record Linkage, Privacy-Preserving Record Linkage, Blocking, Cryptography/Hashing, Recall, Reduction Ratio

Efficient privacy-preserving record linkage (PPRL) is essential for integrating data from different providers without exposing personally identifiable information (PII). This study investigates the effectiveness of a novel blocking technique, implemented on Bloom filters (a space-efficient probabilistic data structure), to enhance efficiency and maintain privacy in a real-world evaluation.

This methodology involves the utilization of Anonlink, an open-source Python-based PPRL system which generates a type of Bloom filter, Cryptographic Longterm Keys (CLK), for secure record linkage. Initially, the relevant PII fields of the two datasets undergo anonymization into CLKs. Utilizing the CLK’s property of similarity preservation, we create manageable blocks of records based on bits in common within the Bloom filters. This allows us to reduce the number of comparisons of non-matching records to improve linkage efficiency. Finally, record linkage is performed to identify potential matches within the blocked datasets.

This blocking technique for CLKs is evaluated in terms of efficiency and record matching precision, aiming to determine the optimal balance between the two factors. Preliminary results indicate a significant reduction in computational burden, with recall minimally affected. Moreover, the implemented blocking technique poses no additional risks of privacy breaches.

Preliminary evaluation of the blocking technique shows a promising avenue for secure and efficient data integration, especially in datasets with PII, warranting further investigation for validation and wider application.

